# Central Nervous Activity upon Systemic Salicylate Application in Animals with Kanamycin-Induced Hearing Loss - A Manganese-Enhanced MRI (MEMRI) Study

**DOI:** 10.1371/journal.pone.0153386

**Published:** 2016-04-14

**Authors:** Moritz Gröschel, Romy Götze, Susanne Müller, Arne Ernst, Dietmar Basta

**Affiliations:** 1 Department of Otolaryngology, Unfallkrankenhaus Berlin, Charité Medical School, Berlin, Germany; 2 Neuroscience Research Center (NWFZ), Charité Medical School, Berlin, Germany; Oregon State University, UNITED STATES

## Abstract

This study investigated the effect of systemic salicylate on central auditory and non-auditory structures in mice. Since cochlear hair cells are known to be one major target of salicylate, cochlear effects were reduced by using kanamycin to remove or impair hair cells. Neuronal brain activity was measured using the non-invasive manganese-enhanced magnetic resonance imaging technique. For all brain structures investigated, calcium-related neuronal activity was increased following systemic application of a sodium salicylate solution: probably due to neuronal hyperactivity. In addition, it was shown that the central effect of salicylate was not limited to the auditory system. A general alteration of calcium-related activity was indicated by an increase in manganese accumulation in the preoptic area of the anterior hypothalamus, as well as in the amygdala. The present data suggest that salicylate-induced activity changes in the auditory system differ from those shown in studies of noise trauma. Since salicylate action is reversible, central pharmacological effects of salicylate compared to those of (permanent) noise-induced hearing impairment and tinnitus might induce different pathophysiologies. These should therefore, be treated as different causes with the same symptoms.

## Introduction

Salicylate is known to induce reversible loss of auditory sensitivity and tinnitus both in humans [1–8] and animals [9–16]. Therefore, the substance has been used in several animal studies that aimed to investigate the generation of tinnitus. Previous *in-vivo* and *in-vitro* studies have shown that acute application of sodium salicylate causes changes of outer (OHC) and inner (IHC) hair cell function, as well as an altered activity in central auditory and non-auditory structures. In the inner ear (the cochlea), salicylate leads to both functional and structural changes. Salicylate markedly reduces the cochlear blood flow by vasoconstriction [17]. Furthermore, changes in IHC synaptic morphology occur [18] with hair cell resting potential modified through a reduction in outward potassium currents [19, 20]. This blocks IHC function, leading in turn to a decreased neurotransmission. In OHCs, vasoconstriction leads to an impaired electro-motility by reducing the lateral wall stiffness [21] and the amount of electro-motile length changes [22–24]. A partial loss of cochlear amplification with its down-regulation of cochlear output is reflected by decreased DPOAE- and CAP-amplitudes. Salicylate action on cochlear NMDA receptors might be partly responsible for peripheral tinnitus generation [13, 25–27].

Beside these cochlear changes, salicylate also modulates neuronal activity in the central auditory pathway in a dose-dependent manner. It has been demonstrated that changes in cochlear output leads to an altered spontaneous activity in the auditory nerve. Whereby salicylate studies in cats revealed an increase in nerve fibre activity, spontaneous discharge rates were shown to decrease in gerbils [28, 29, 30]. Histological studies have shown that salicylate increases c-fos expression in the dorsal cochlear nucleus (DCN), inferior colliculus (IC), medial geniculate body (MGB) and auditory cortex (AC) [31, 32]. However, an elevation was also observed in several non-auditory structures in the brainstem, thalamus or amygdala [31, 33]. Furthermore, an enhanced activation of neural tissue has been shown in the ventral cochlear nucleus (VCN), indicated by an increased neuronal nitric oxide synthase (nNos)-expression in principal neurons [34]. Salicylate also causes increased activity in serotonergic neurons, accompanied by an altered synaptic function, resulting in a downregulation of GABA and an increase in glutamate activity in tinnitus-related auditory brain structures [9, 35–40]. An upregulation of the genes Arc/Arg3.1 and Egr-1, as well as the NMDA receptor subunit 2B (NR2B) in the dorsal cochlear nucleus, indicates a strengthening of central excitatory synaptic connections due to salicylate application [41]. The changes observed in neurotransmission may induce sound-evoked hyperactivity due to an increase in excitatory projections [42–44], particularly affecting the thalamo-cortical system during tinnitus generation [27, 45]. Further, it has been shown that cochlear output and AC tonotopy are shifted towards tinnitus-associated frequencies [46].

Beside these findings, several previous studies have investigated the effects of systemic salicylate application with regard to the modulation of spontaneous or evoked activity in central auditory structures. In summary, the results demonstrated that the majority of neurons change their spontaneous activity upon salicylate application. An increase was observed in the frequency range showing tinnitus-related behaviour, whereby neurons with low spontaneous firing rates were particularly affected, even in the auditory cortex. In contrast, a salicylate-induced reduction in multi-unit activity was also detected in awake animals (e.g.: IC–[47–49]; AC–[27, 44, 50, 51]). Moreover, spontaneous calcium-dependent activity, after systemic salicylate application, was significantly increased at the level of the dorsal cochlear nucleus (DCN) and inferior colliculus (IC) [52]. When measuring evoked neuronal responses after salicylate treatment, a depression of cochlear potentials has been observed, whereby local field potentials were increased in central nervous system structures, including medial geniculate body, auditory cortex and lateral amygdala [53].

These changes of central neural activity could be caused by the peripheral or the central effects of salicylate. Recordings in brain slices showed a direct, dose-dependent pharmacological action of salicylate on several central auditory and non-auditory structures [54, 55]. However, little is known about the impact of central and peripheral effects of salicylate application on the generation of salicylate-induced tinnitus.

Using manganese-enhanced magnetic resonance imaging (MEMRI), the present study investigated the effect of systemic salicylate application on calcium-related activity in central auditory structures after reducing the peripheral effects mediated by cochlear hair cells as one major target of salicylate action.

## Materials and Methods

### Animals

In the present study, 30 normal hearing adult (postnatal 30–60 day) mice (*Mus musculus*, NMRI strain) of both sex were examined. Different animals were used for threshold testing and MEMRI measurements. The experimental protocol was approved by the governmental commission for animal studies (LaGeSo Berlin, approval number: G 0153/06). Experiments were carried out in accordance with the EU Directive 2010/63/EU on the protection of animals used for scientific purposes. All efforts were made to minimize the number of animals and their suffering. The condition of animals was monitored every 6 hours after the induction of hearing loss and, in addition, every hour following substance injection (kanamycin, salicylate or manganese, respectively). Since animals did not show any abnormal behaviour, additional analgesic treatment was not necessary.

### Experimental induction of kanamycin-induced hair cell damage

For 22 mice, cochlear tissue was damaged by a subcutaneous injection of kanamycin sulfate (Carl Roth, Karlsruhe, Germany) dissolved in phosphate-buffered saline (PBS), at 1 mg/g body weight followed 40 minutes later by an intraperitoneal injection of bumetanide (Sigma-Aldrich, St. Louis, MO, USA) at 0.05 mg/g body weight. This procedure has been shown to elicit a significant and rapid hair cell loss in mice *in-vivo* [56]. Three of the above treatments were administered, each separated by 48 hours, to ensure a profound loss of sensory outer and, to a lesser extent, inner hair cells. Following kanamycin and bumetanide treatment, 7 animals underwent ABR recordings while 15 mice were used for MEMRI measurements.

### ABR measurements

Auditory thresholds were measured for 7 hearing-impaired mice (48 hours after the final injection) and for 8 normal hearing control mice. Different animals were used for each group. The time interval for deaf mice was chosen to avoid any additional central pathophysiological effects arising from the damaged peripheral auditory system.

Frequency-specific (4, 8, 12, 16 & 20 kHz) auditory brainstem responses (ABR) were recorded under anaesthesia. Sub-dermal needle electrodes were placed at the vertex (active), mastoid (reference) and at one foot (ground). Tone stimuli were delivered binaurally at different SPLs with a sine-wave generator (Modell SSU2, Werk für Fernmeldewesen, Berlin, Germany). ABR recordings were carried out using a Viking IV^®^ measurement system (Viasys Healthcare, Conshohocken, Pennsylvania, USA). The brainstem responses (recording epoch 10 ms following stimulus presentation) were amplified (100.000 x), filtered (bandwidth 0.15–3 kHz) and averaged (300 x) by the Viking IV^®^. The amplitude growth function of wave IV/V was calculated from the resulting data for each frequency tested and a linear regression was fitted to the function. By extrapolating the regression line to zero, mean auditory thresholds were estimated for the controls and the kanamycin-treated animals. Results are shown as mean auditory thresholds (± S.E.) in dB SPL by group and mean relative hearing loss of the treated group compared to the normal hearing controls.

### Salicylate application

To investigate salicylate-induced changes in central calcium-dependent activity after kanamycin-induced damage of cochlear hair cells, 9 treated animals received an intraperitoneal (i.p.) injection of a sodium salicylate (SS) solution (250 mg/kg). It has been shown earlier that salicylate is able to cross the blood brain barrier shortly after systemic (e.g. i.p.) application leading to an increased cerebrospinal fluid level of salicylate [57–59]. Comparable SS concentrations were also used in former studies to elicit salicylate-induced tinnitus in rodents [13, 60, 61]. SS was injected 48 hours after the last kanamycin/bumetanide treatment.

### Manganese-enhanced magnetic resonance imaging (MEMRI)

MEMRI is a powerful tool to image central nervous system activity in small animals *in-vivo* [62–64]. After systemic application of a manganese chloride solution, manganese ions cross the blood-brain barrier [65] and enter excited cells by substituting calcium influx during neuronal activity [64, 66, 67]. Due to a slow clearance, Mn^2+^ accumulates within the tissue which results in an increase in MRI-T1 signal contrast and therefore reflects the Ca^2+^-dependent activity [68]. Thereby, neuronal activity is monitored using the MEMRI technique and, thus, Ca^2+^-dependent activity can be imaged. This provides an opportunity to integrate neuronal activity, represented by the increase in signal contrast due to manganese accumulation, over a well-defined period of time before measurements, i.e., 24 hours in the present experiments. This is of particular importance during the investigation of auditory-related activity even inside a noisy MRI scanner [52, 69–71].

On the day of the experiments and 48 hours after the final kanamycin/bumetanide treatment (i.e. immediately after SS injection in the salicylate group), animals of the experimental group (treated with kanamycin/bumetanide and salicylate) as well as hearing-impaired control mice (kanamycin/bumetanide-treated only) received a 0.4 mM/kg dose of MnCl_2_ solution (in accordance with [71]). Delivery was via a single intraperitoneal injection. Twenty-four hours after the manganese treatment (prior to this animals were kept in their cages in a quiet environment), when manganese accumulation reached its maximum level in the relevant brain structures [72], MRI scanning was performed. During MRI scanning, anaesthetised mice were placed on a heated circulating water blanket to ensure constant body temperature of 37°C. Anaesthesia was induced with 3% and maintained with 1.5–2.0% isoflurane (Forene, Abbot, Wiesbaden, Germany) delivered at 0.5 l ⁄ min of 100% O2 via a facemask under constant ventilation monitoring (Small Animal Monitoring & Gating System, SA Instruments, Stony Brook, New York, USA). Animals were euthanized after MRI measurements by an overdose of isoflurane (>5% isoflurane at 0.5 l/min of 100% O2).

**Fig 1:**
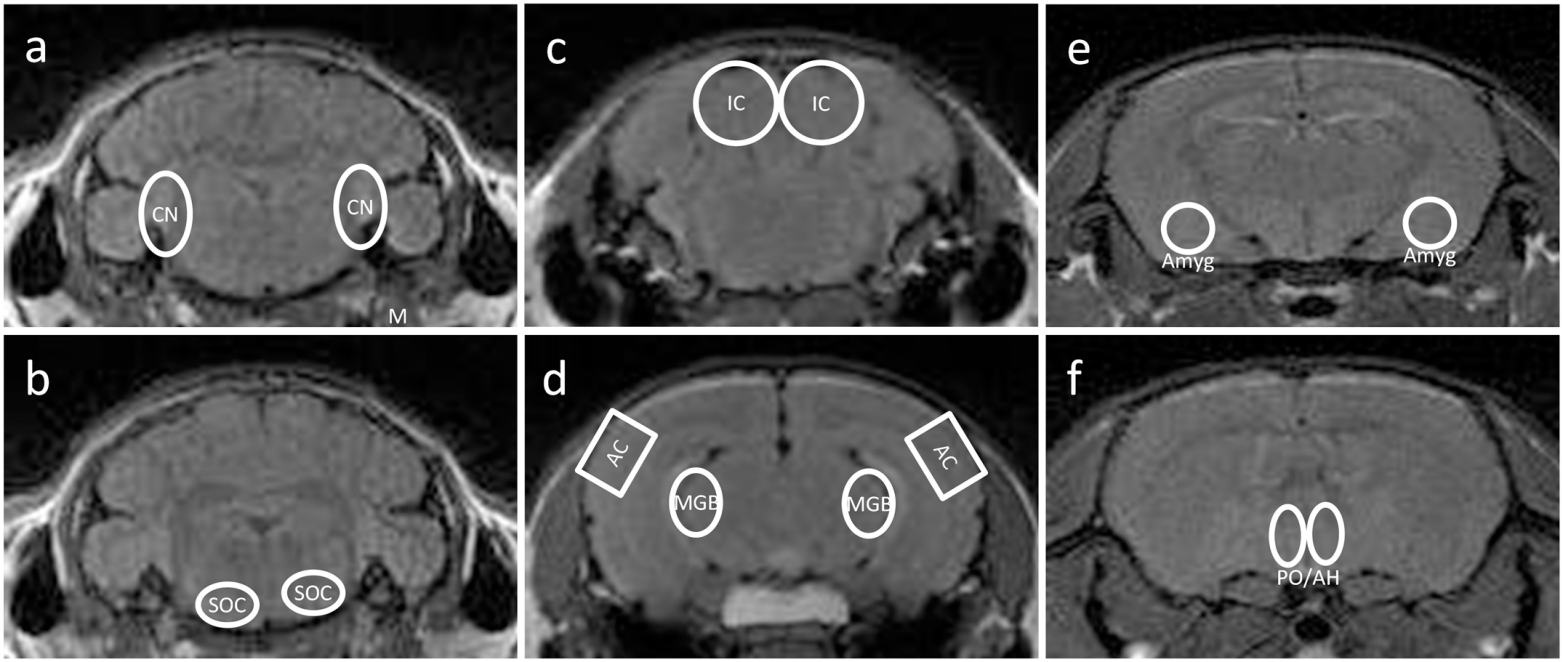
Examples of MEMRI-images of a mouse brain with labelled regions of interest. Outlines show the auditory structures of: a) the dorsal and ventral cochlear nucleus (CN), b) the superior olivary complex (SOC), c) the inferior colliculus (IC), d) the medial geniculate body (MGB) and primary auditory cortex (AC). Further, the non-auditory structures of e) the amygdala (Amyg) as well as f) the preoptic area of the anterior hypothalamus (PO/AH) are indicated in the images. Masseter muscle (M) is indicated in image a) on the CN level, used for normalization of image brightness within each slice. (Images were taken from a preliminary study to establish the MEMRI method performed on untreated animals).

Manganese-enhanced MRI was carried out using a 7 Tesla rodent scanner (Pharmascan 70 / 16AS, Bruker BioSpin, Ettlingen, Germany) with a 16 cm horizontal bore magnet and a 9 cm (inner diameter) shielded gradient with a H-resonance-frequency of 300 MHz and a maximum gradient strength of 300 mT/m. For imaging a 1H-RF quadrature-volume resonator with an inner diameter of 20 mm and a T1-weighted 2D turbo spin-echo sequence (TR / TE = 938 / 10.6 ms, RARE factor 2, 6 averages) was used. Data acquisition and image processing were conducted using the Bruker Paravision 4.0 software. Thirty-five axial slices with a slice thickness of 0.3 mm, a field of view of 2.85 x 2.85 cm and a matrix of 256 x 256 resulted in an in-plane resolution of 111μm. Imaging covered the brain from brainstem to forebrain. Signal intensity analysis of defined auditory brain regions was carried out with the program Analyze 5.0 (AnalyzeDirect, Inc.; Lenexa USA). Signal intensities were quantified bilaterally in the dorsal (DCN) and ventral (VCN) cochlear nucleus, the superior olivary complex (SOC), the inferior colliculus (IC), the medial geniculate body (MGB), the auditory cortex (AC), the preoptic area of the anterior hypothalamus (PO/AH), the Amygdala (Amyg). The masseter muscle was used as an objective intensity reference, since it is located close to the related neural tissue but is not affected by changes in brain activity. PO/AH and Amyg served as non-auditory structures investigated in the present study. Regions of interest were marked in accordance with “the mouse brain atlas in stereotaxic coordinates” [73] (Fig 1). The investigated brain structures were manually outlined within each relevant brain slice and a voxel-based analysis was carried out for each structure under observation. Due to variations in each animal’s position within the MRI scanner, the dimensions of each brain structure (the tissue volume) in the scan could vary across subjects. Therefore, the number of voxels included in each structure differs slightly. Signal intensities for all analysed structures were normalized in relation to the intensity of the muscle at the same side. The relative MRI signal (given in normalized relative units) was calculated for each animal by using the mean of the measured signal strength of every slice for each analysed structure, normalized in relation to the intensity for the muscle at that side. The results of all animals were compared between the salicylate group and the hearing-impaired control group for each investigated brain area.

### Statistical analysis

The statistical comparison was done with the software SPSS (IBM SPSS Statistics Version 20, IBM Corp., Armonk, New York, USA). After confirming a normal distribution using the Kolmogoroff-Smirnoff-test, the t-test for independent samples was applied. A statistically significant difference was considered for p<0.05.

## Results

### ABR-recordings

**Fig 2:**
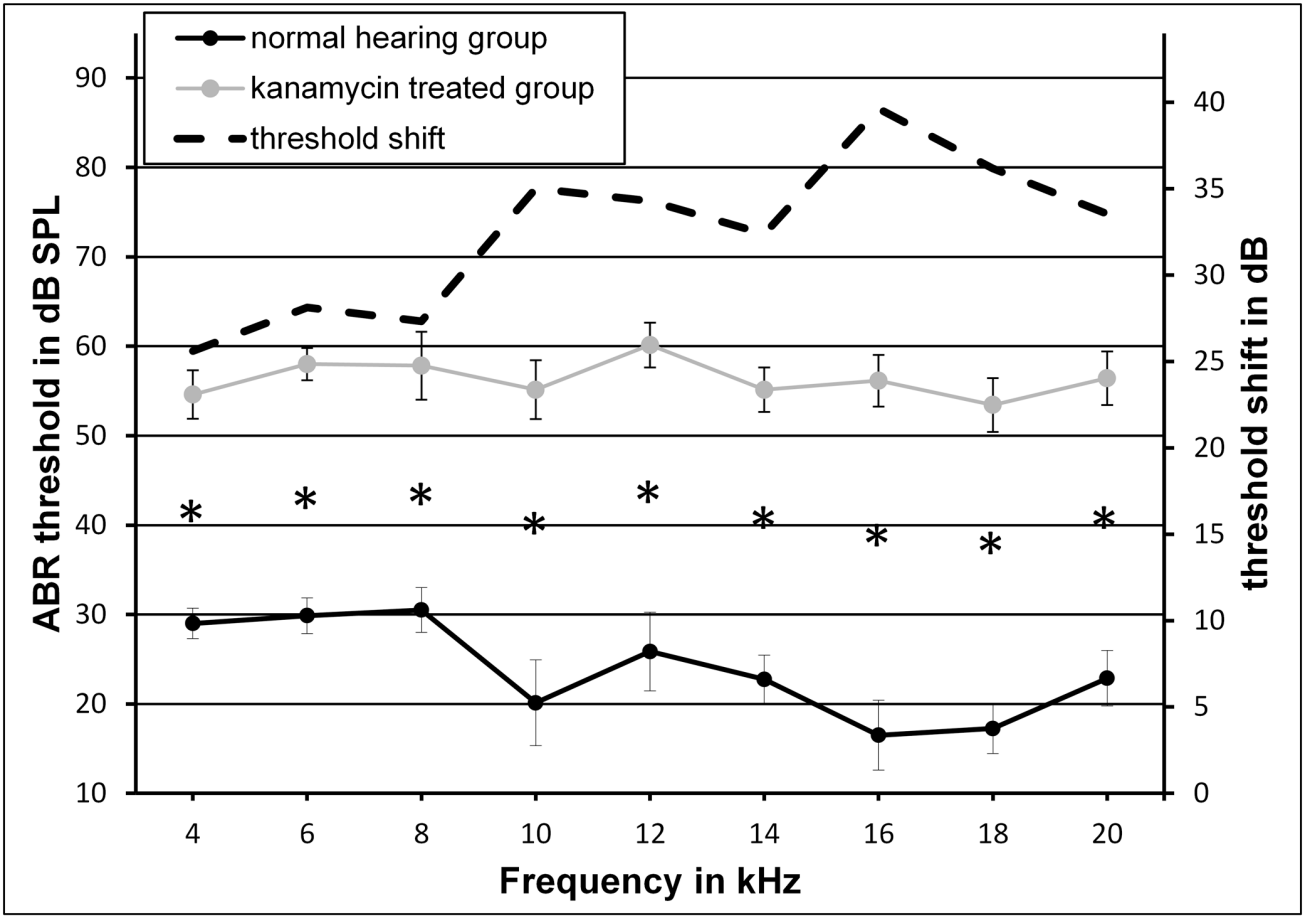
Auditory thresholds before and after damage of the auditory periphery. Hearing thresholds (mean±S.E.) in mice 48 hours after a threefold injection (applied every 48 hours) of kanamycin (1 mg/g body weight) and bumetanide (0.05 mg/g body weight) (kanamycin-treated group, n = 7) in relation to normal hearing control animals (normal hearing group, n = 8). Graph further indicates the mean threshold shift of the experimental group. Asterisks point to significant differences between normal hearing and hearing impaired animals for all investigated frequencies between 4 and 20 kHz (p<0.001).

Hearing loss following kanamycin and bumetanide treatment was indicated by an ABR-threshold shift of up to 40 dB in the investigated frequency range between 4 and 20 kHz compared to the untreated normal hearing control group. Frequency-dependent mean auditory thresholds in normal hearing controls ranged between 17 and 31 dB SPL. In the hearing impaired animals, thresholds were detected between 53 and 60 dB SPL (Fig 2). Average hearing loss in the experimental group ranged between 26 dB at 4 kHz and 40 dB at 16 kHz. However, threshold shift was highly significant for all frequencies (p<0.001).

### MEMRI

Following systemic salicylate application, MEMRI contrast increased significantly in all investigated auditory brain areas.

**Fig 3:**
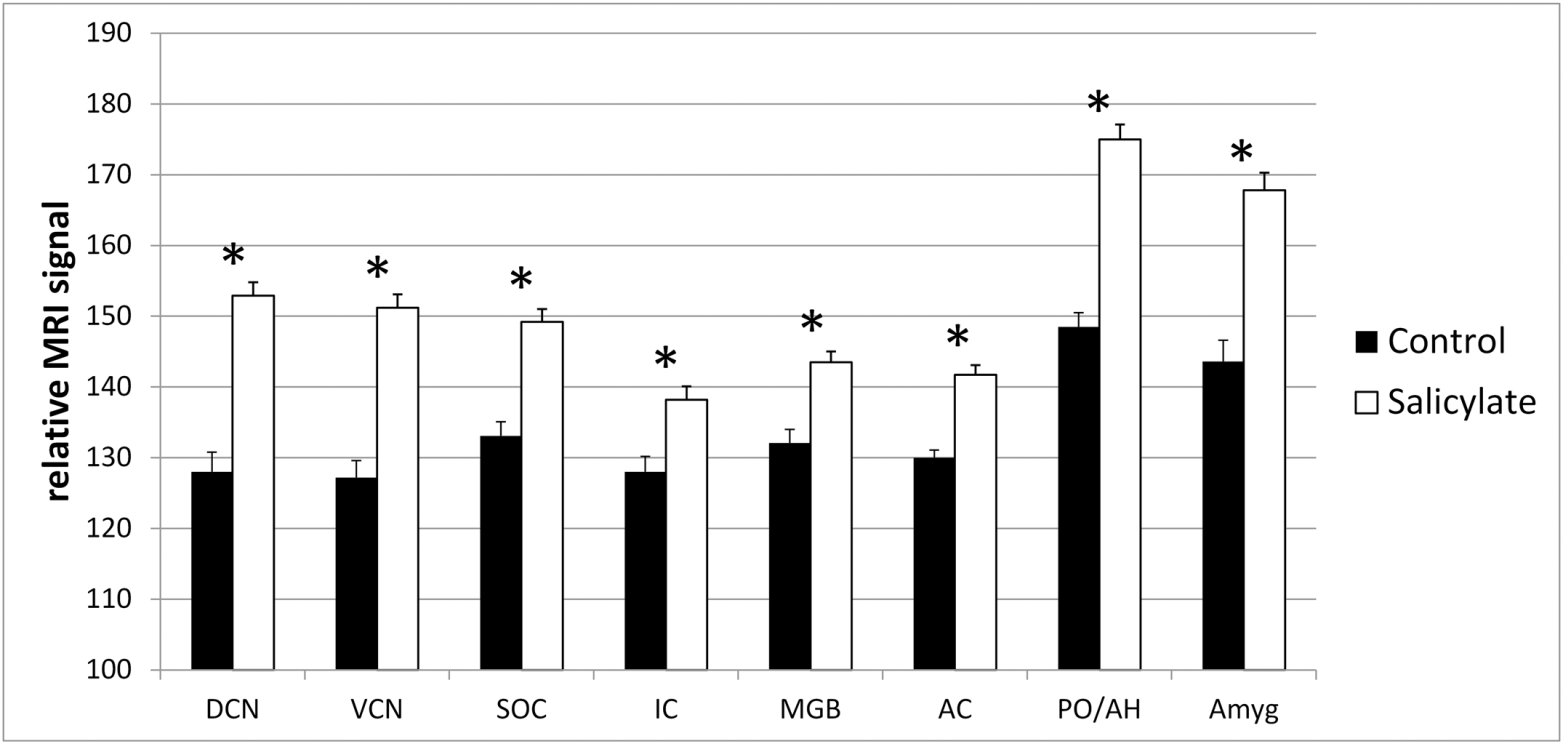
Mean MEMRI contrast of experimental groups. Relative manganese-enhanced MRI-T1-contrast (mean±S.E.) for the investigated auditory and non-auditory brain structures in hearing-impaired control (black columns) compared to salicylate-treated (white columns) mice. Both groups had a profound hearing loss due to peripheral damage by kanamycin/bumetanide treatment before experiments. Asterisks indicate significant differences between the groups (p<0.05). Investigated structures: dorsal cochlear nucleus (DCN), ventral cochlear nucleus (VCN), superior olivary complex (SOC), inferior colliculus (IC), medial geniculate body (MGB), auditory cortex (AC), preoptic area of the anterior hypothalamus (PO/AH), Amygdala (Amyg).

In detail, the relative MRI signal intensities were raised in the DCN from 128.0±2.8 in hearing-impaired controls to 152.9±1.9 in the hearing-impaired salicylate group and for the VCN from 127.2±2.4 (hearing-impaired controls) to 151.2±1.9 (salicylate group). In the SOC, the signal increased from 133.1±2.0 (in hearing-impaired controls) to 149.2±1.8 in salicylate-treated animals. Similar observations have been made in the IC (hearing-impaired controls: 128.0±2.2; salicylate group: 138.2±1.9), in the MGB (hearing-impaired controls: 132.1±1.9; salicylate group: 143.6±1.5) as well as in the AC (hearing-impaired controls: 130.0±1.1; salicylate group: 141.7±1.4) (Fig 3).

An increased MEMRI signal was also observed for the non-auditory control areas (PO/AH and Amyg) after salicylate treatment. Signal intensities were elevated from 148.5±2.0 (hearing-impaired controls) to 175.0±2.1 (salicylate group) in PO/AH and from 143.6±3.0 in hearing-impaired controls to 167.8±2.5 in salicylate-treated animals in the amygdala region (Fig 3). All data are given in normalized relative units (calculated from the measured signal intensities, as described in the methods section) as mean ± S.E.

The differences between the groups were statistically significant for all investigated auditory and non-auditory structures (p≤0.001 for all comparisons).

## Discussion

The results of the present study demonstrate changes in calcium-dependent brain activity after systemic salicylate application in kanamycin-treated animals using non-invasive manganese-enhanced magnetic resonance imaging (MEMRI).

### ABR thresholds

The ABR recordings demonstrated that a three-fold application of kanamycin and bumetanide produces a significant threshold shift in mice, possibly in particular due to OHC destruction according to previous reports. It is further assumed that the treatment induces a minor loss of IHC and spiral ganglion cells as well [56, 74]. Although it has not been directly investigated here, such a decrease of sensory cells is supported by the electrophysiological findings presented here. The hearing loss after kanamycin and bumetanide treatment of up to 40 dB matches to the results of other studies showing that a near-complete loss of OHC function in mice results in a comparable shift of auditory thresholds [26]. These findings lead to the assumption that the observed central effects of salicylate are only marginally related to its action on outer hair cells. Inner hair cells might also be affected which could further contribute to our results.

### Ca^2+^-related effect of salicylate after peripheral hearing impairment

Recent studies have reported that the central auditory system largely contributes to the salicylate-induced effects on neuronal activity and auditory phantom perceptions like tinnitus, even after disconnection of the auditory periphery [54, 75, 76]. In the present data, the calcium-related neuronal activity was increased after systemic application of a sodium salicylate solution in several investigated brain structures. This followed kanamycin-induced reduction of the peripheral input from cochlear sensory cells. In addition, it was shown that the central effect of salicylate is not limited to the auditory system, but shows a rather general alteration of calcium-related activity, as indicated by the increased manganese accumulation in the preoptic area of the anterior hypothalamus (PO/AH) as well as in the amygdala. Basta et al. [55] showed an increased spontaneous activity during salicylate superfusion in brain slices of auditory and non-auditory (hypothalamic) structures. The hypothalamus is a central structure responsible for thermoregulation, which maintains the body temperature at a constant level. When salicylate is given in patients with elevated temperature (fever), it exerts an antipyretic effect. In rats, the intra-ventricular application of salicylate close to the PO/AH can induce hypothermia [77], which might be related to an altered neuronal activity in the corresponding structure as shown in the data (body temperature was kept constant during MRI scanning only). This additional activity increase, despite the overall changes in neural tissue, could also provide an explanation for the higher salicylate-induced effect in the hypothalamus and amygdala compared to the auditory system. Further support comes from earlier findings that systemic application of salicylate leads to a strong decline in body temperature [78].

An altered activity of the amygdala after salicylate application has already been reported by immunostaining. An elevated c-Fos- or arg3.1-related activity was demonstrated in the auditory cortex and the amygdala after treatment with a single high-dose salicylate application [33], whereby a decrease was observed in VCN and IC. In the present study, an increase in MEMRI signal intensity within the amygdala and therefore, an elevation in calcium-related activity, might rely on similar mechanisms responsible for synaptic transmission. However, further investigation is needed to clarify the differences in subcortical auditory structures.

One possible mechanism for this increase in calcium-related activity could be the direct action of salicylate on neuronal and synaptic activity within those particular brain structures. Salicylate largely influences the pre- and postsynaptic conductance of several transmembrane ion channels by modulating neurotransmitter activity (e.g. modulation of GABA-mediated inhibition) [38, 40]. In turn, the activation of neurons by releasing action potentials and increasing excitatory synaptic transmission can be induced [37, 79–81]. Further, an increase in immediate early gene activity and its possible function in upregulation of NMDA receptor activity was demonstrated in the dorsal cochlear nucleus [41]. These mechanisms depend on intracellular calcium and might induce calcium and manganese influx into the cell, both being able to elicit several calcium-related neuronal responses [65, 66, 82]. This might have contributed to an elevated intracellular manganese accumulation followed by a higher MEMRI signal intensity in the present data. This idea is supported by our recent findings of salicylate effects in brain slices [55].

It should be mentioned that pharmacological removal of cochlear sensory cells reduces auditory nerve input towards the central auditory system. This happens particularly when inner hair cells have been affected (in addition to OHC damage), thereby inducing changes in central neuronal activity (e.g. compensatory hyperactivity), as shown by several studies in this field. For a review, see [83]. However, hearing impaired controls as well as salicylate-treated animal should be affected to a similar extent. Another important issue is that salicylate application itself has the property to further diminish sensory function. It has been reported that salicylate particularly influences outer hair cell motility and thereby significantly reduces stimulus-driven auditory nerve activity [22, 46, 84, 85]. Any such effect should play only a minor role in the present data, since spontaneous calcium-related activity was measured and animals were kept in a quiet environment to prevent external acoustic stimulation during the experiments. However, with the current experimental design, an additional salicylate-induced hearing impairment and a further salicylate-driven effect on the function of surviving hair cells could not be excluded completely and might therefore slightly contribute to the observed effects.

The present study indicates that salicylate-induced activity changes in central auditory brain areas, including possibly tinnitus, exist to a large extent. Since observations were made after reduction of peripheral effects mediated by cochlear hair cells, these effects cannot be entirely explained by salicylate’s action on peripheral tissue.

### Is there a correlation with noise-induced hearing loss/tinnitus?

The present results indicate that the mechanisms through which salicylate impacts on activity in several auditory and non-auditory brain structures appears different to those known from noise-induced hearing loss (and possibly tinnitus generation); particularly immediately after treatment. Acute noise-exposure is accompanied initially by cochlear changes. In a second stage, the altered afferents are probably responsible for modulating central auditory processing. However, permanent tonotopic and other pathophysiological changes appear to be largely established by compensatory neuroplasticity, acting to overcome the reduced cochlear output and damaged central auditory structures. This is in line with the finding that an increased spontaneous firing rate (SFR) in the DCN (after 140 dB SPL noise exposure) could be recorded only after a 2–3 days’ period [86]. Once these changes are established as autonomous nervous activity, they seem to become uncoupled from cochlear projections and show a rebalancing of central inhibitory and excitatory neurotransmission [87, 88]. It has been demonstrated that sectioning of the auditory nerve and, thus, abolishing the peripheral input, had no effect at all on the increased SFR in the DCN after noise exposure [89]. Further discrepancies between the impact of noise and salicylate became apparent when comparing the current data with our recent findings using the MEMRI technique. In earlier MEMRI experiments we demonstrated that noise-induced hearing loss leads to short- and long-term changes in calcium-related activity in central auditory structures [70]. As the generation of tinnitus was not tested in both studies, differences in Ca^2+^ activity patterns between the two studies even indicate that hearing impairment related to either salicylate application or noise trauma have varying impacts on central auditory system physiology and might therefore, rely on different pathophysiological mechanisms. This may lead to the hypothesis that the appearance of tinnitus due to salicylate or noise is also based on different sources. Calcium-related activity patterns have also been observed by another study investigating activity changes in several brain structures after induction of noise- or salicylate-related tinnitus using manganese-enhanced MRI [52]. Signal intensity differences between noise and salicylate treatment were only present in some of the structures investigated: e.g. the DCN and part of the inferior colliculus (DCIC). The discrepancies between these studies might be related to the particular experimental design, as Holt and colleagues used rats and their animals were normal hearing before treatment. Moreover, noise was delivered unilaterally and was narrow-band, which is in contrast to our recent work and could explain the different results.

In essence, the present data support the idea that the impact of salicylate application or noise trauma on the auditory system are probably based on different mechanisms, even if comparable changes of electrophysiological properties could be observed in vivo: e.g. increased SFR in central auditory areas [47, 90, 91], alterations in synaptic transmission [9, 40, 41, 92, 93] and an increase in neural synchrony [51, 94]. Since salicylate action is reversible, central pharmacological effects of salicylate compared to those of (permanent) noise-induced hearing impairment and tinnitus seem to induce different pathophysiologies and might therefore, be considered as different causes for the same symptoms.

## Supporting Information

S1 Table Raw data used in ABR analyses. (XLS)

S2 Table Raw data used in MEMRI analyses. (XLS)
